# The role of myeloid-derived suppressor cells in children

**DOI:** 10.3389/fped.2025.1525143

**Published:** 2025-02-27

**Authors:** Jordan Brauner, Anna Wilt, Christopher P. Montgomery, Katherine Bline

**Affiliations:** ^1^ Department of Pediatrics, Nationwide Children’s Hospital, Columbus, OH, United States; ^2^ University of Minnesota Health Sciences, University of Minnesota Medical Center, Minneapolis, MN, United States

**Keywords:** pediatric, myeloid-derived suppressor cells (MDSCs), T cell, immune suppression, NK cell

## Abstract

Myeloid-derived suppressor cells (MDSC) were first recognized over twenty years ago as a key immunomodulatory cell population. Since their initial identification, a growing body of literature points to the importance of MDSC as a heterogeneous, immunosuppressive cell population and as a therapeutic target in adults with cancer. MDSC are potent suppressors of T cells and Natural Killer (NK) cells and can be helpful or harmful to the host depending on the pathophysiology. For example, MDSC are beneficial in pregnancy and prevent spontaneous abortion by promoting maternal-fetal tolerance. Increased MDSC are also associated with improved outcomes in patients with graft vs. host disease by decreasing T cell-driven inflammation. However, MDSC can also be harmful and are known to be pathologic in adults with cancer and chronic infections by promoting tumor escape and impairing pathogen clearance, respectively. Despite the widespread recognition of the importance of MDSC and their immune suppression effects in adults, much less is known regarding the role of MDSC in children. Research investigating MDSC in children lags significantly behind adult studies. In fact, while over 5,000 publications on PubMed discuss MDSC in immune regulation, fewer than 50 of these publications focus specifically on their role in children. This review aims to summarize the existing literature on the role of MDSC in children and identify important directions for future research, including targeting these cells in the pediatric population to improve clinical outcomes.

## Introduction

The innate and adaptive immune systems work together to determine the host response to foreign pathogens and injury. As the first line, the innate immune system mounts a rapid response, relying on epithelial cells, phagocytes, natural killer cells, dendritic cells, and the complement system. In contrast, the adaptive immune system takes longer- ranging from hours to day- to generate a more specific and substance response. A key feature of adaptive immunity is immunological memory, which enables a faster and more effective response upon pathogen exposure. This long-term protection is driven by two types of lymphocytes: antibody-producing B cells, and T cells, which mediate cellular responses (1). Adaptive immunity also includes myeloid cells, primarily phagocytes, antigen presenting cells, and mast cells (1). Collectively, these cells create an orchestrated response that ensure effective pathogen clearance while preventing excessive damage to the host. This balance is achieved through an initial inflammatory response that eliminates the pathogen while counterregulatory mechanisms simultaneously limit inflammation to protect healthy tissues. A key component of this regulation is immunosuppressive MDSC, which are crucial for preventing hyperinflammation in response to pathogens.

MDSC are a heterogenous cell population of immature myeloid progenitors that expand in response to inflammation and are defined by their ability to potently suppress T cells (2–4). MDSC originate from the bone marrow and comprise about 2% of the circulating peripheral blood mononuclear cell population in healthy adults (5, 6). Although first described decades ago, a uniform nomenclature in the literature for human-derived MDSC remained elusive for many years. In 2007, Gabrilovich et al. recommended that these cells be named myeloid-derived suppressor cells to encompass both their origin and functional activity and provide consistency in published literature (3). MDSC can be identified by flow cytometry using the cell surface markers CD45^+^, HLA-DR^−^, CD33^+^/CD11b^+^ (2, 4, 7–9). There are two major subsets of MDSC: polymorphonuclear or granulocytic MDSC (G-MDSC), which resemble immature neutrophils, and monocytic MDSC (M-MDSC), which resemble monocytes (2, 4, 6, 7, 9–11). G-MDSC are identified by cell surface markers CD14^−^/CD15^+^ in humans and express high levels of reactive oxygen species (ROS) but low levels of nitric oxide (NO) (2, 4, 9). In contrast, M-MDSC are identified by cell surface markers CD14^+^/CD15^−^ and express high levels of NO with low levels of ROS (2, 4, 9). Both subsets can express programmed death-ligand 1 (PD-L1) and arginase-1 (Arg-1) (4, 9, 12). A third subset, termed early-stage MDSC (E-MDSC), are less well characterized and defined as immature, precursor MDSC comprised of HLA-DR^−^ CD33^+^/CD11b^+^ but CD14^−^/CD15^−^ cells in humans (2). While potent T cell suppression is universal to all MDSC subtypes, evidence indicates the subtype predominance and function may be pathology-dependent (4, 6, 13).

Initially described in cancer research, more recent evidence shows that MDSC expansion occurs in other inflammatory conditions, including infections, trauma, autoimmune diseases, and organ transplantation (4). During these conditions, myeloid cells fail to differentiate into mature granulocytes, macrophages, or dendritic cells, leading to an expansion of the immature MDSC population (4, 8, 10). MDSC expansion is driven by various cytokines and growth factors, including proinflammatory IL-6 and GM-CSF (14, 15). These signals activate intracellular pathways that converge on the Janus kinase (JAK) family and the transcription factors known as signal transducers and activators of transcription (STAT) (4). In particular, STAT3 upregulates myelopoiesis and inhibits the differentiation into mature myelocytes to promote MDSC expansion (4). Recent studies indicate that MDSC must be activated to effectively suppress T cells. Cytokines such as INF-ɣ and IL-4, produced by activated T cells and tumor stromal cells, drive this activation by inducing Arg-1 and NO expression (4).

The defining characteristic of MDSC is their potent inhibition of T cell activity and/or proliferation (2, 4, 8, 10). A primary mechanism of MDSC-mediated suppression is the increased production of Arg-1, which depletes the microenvironment of arginine, an essential amino acid required for CD3/CD28 T cell receptor function and T cell proliferation (4, 8, 9). Additionally, MDSC can upregulate inducible nitric oxide synthase (iNOS) and ROS, generating free radicals that lead to impairment of T cell function (4, 6, 9). MDSC also promote the expansion of T regulatory cells, an immunosuppressive CD4+ T cell subset (16).

**Figure 1:**
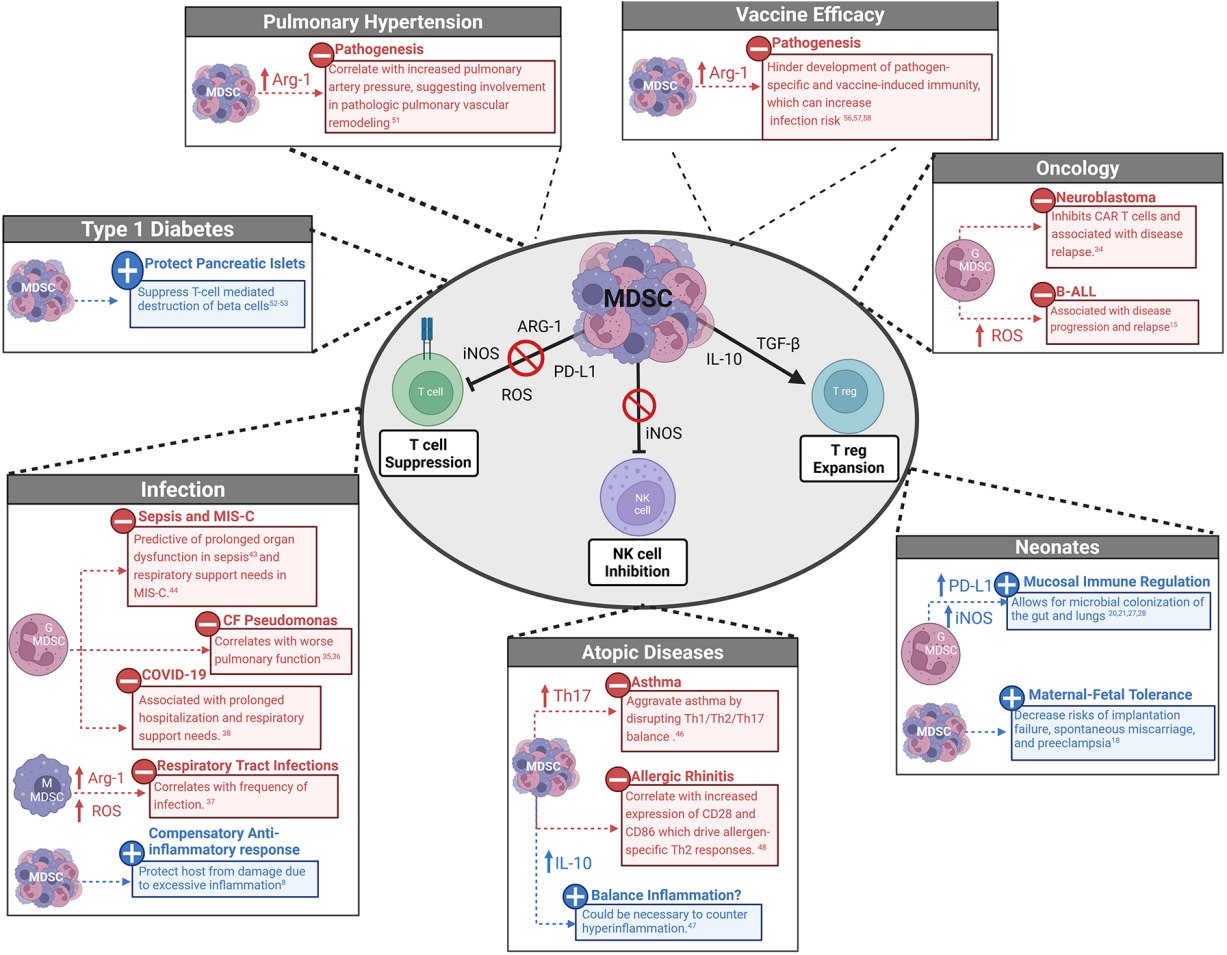
Dichotomous role of and mechanisms of MDSC suppression in children. Myeloid-derived suppressor cells (MDSC) can suppress Natural Killer (NK) cells and T cells and promote expansion of T regulatory cells (T regs) via direct cell contact and production of cytokines and enzymes. MDSC can have a beneficial or harmful role depending on the pathophysiology of pediatric disease or neonatal immune development. MDSC, Myeloid- derived suppressor cells; G-MDSC, granulocytic-MDSC; M-MDSC, monocytic- MDSC; ARG-1, arginase-1; iNOS, inducible nitric oxide synthase; ROS, reactive oxygen species; PD-L1, programmed death- ligand 1; TGF-β, transforming growth factor β, NK, natural killer; T reg, T regulatory cells; B-All, B cell acute lymphoblastic leukemia; Th, T helper; IFN, interferon; MIS-C, multisystem inflammatory syndrome; CF, cystic fibrosis; COVID-19; coronavirus disease 2019; CAR, chimeric antigen receptor.

MDSC have been identified in a variety of pathologies, where their effects can be beneficial or harmful depending on the context. They are well characterized in adults with malignancies, and results from clinical trials suggest that inhibition of MDSC in adults with cancer decreases tumor burden and improves patient outcomes (17). Although less is known about MDSC in pediatric patients, recent evidence suggests that these cells are a potential therapeutic target with similarly dichotomous roles in children (Figure 1). This review will focus on the role of MDSC in pediatric disease pathology.

### Neonates

MDSC play a crucial role in regulating immune responses during pregnancy and the neonatal period. In utero, increased MDSC in pregnant women and in neonatal cord blood are associated with decreased risk of implantation failure, spontaneous miscarriage, intrauterine growth restriction, and preeclampsia (18). The neonatal immune system is inherently tolerant, preventing harmful interactions with the maternal host before birth and facilitating acclimation to the external environment following delivery. In newborns, MDSC help prevent a hyperinflammatory response as infants encounter nutritional antigens and the gut becomes colonized with microbiota. Higher MDSC levels are associated with a reduced risk of necrotizing enterocolitis, likely by promoting immune tolerance as the intestinal mucosa develops a biofilm (19). MDSC are also present in breast milk, where they correlate with gestational age and contribute to neonatal mucosal immunity (20, 21).

However, the immature and more tolerant immune system in the neonatal period also places this population at higher risk of severe infections. It is hypothesized that persistent expansion of MDSC contributes to an increased risk of neonatal sepsis, a leading cause of mortality in this patient population (22–25). This relationship was explored in a 2018 study by Schwartz et al, who found an increase in G-MDSC in both cord and peripheral blood of preterm infants, regardless of gestational age (23). Interestingly, G-MDSC were greater in preterm infants with underlying infection, including neonatal sepsis and intra-amniotic infection, compared to those without infection. The percentage of G-MDSC expansion also positively correlated with white blood cell counts and C-reactive protein levels in infected infants (23). Lieber and colleagues demonstrated that G-MDSC from cord blood exposed to *E. coli* had increased expression of the cell surface marker PD-L1 and potently suppressed T cell proliferation (26). Although G-MDSC maintained their phagocytic capabilities, they secreted cytokines with a skewed anti-inflammatory profile compared to mature neutrophils, which produced more pro-inflammatory cytokines (12). Although MDSC are necessary for the development of the gut microbiome in the neonate (19), these cells may become pathological during acute infection. Further investigation into MDSC as a potential mechanism and therapeutic target in neonatal sepsis could help to improve outcomes for a disease process that is a leading cause of mortality in the neonatal population.

Additionally, there are several mouse models looking at a role of MDSC in pregnancy (27–29). One study demonstrated a role for MDSC in immune regulation during the establishment of the neonatal microbiome where mice with reduced G-MDSC showed increased T cells and alterations in the commensal gut bacteria compared to wild type mice (29). Another study supported the role of MDSC in immune tolerance to pregnancy and indicated the A2BR (adenosine 2B receptor) on myeloid cells could be a possible mechanism of T cell suppression necessary for maternal-fetal tolerance. (27) Finally, additional work indicated a role for MDSC in both peripheral T cell homeostasis and T cell development in the thymus (28). Overall, these studies support the role of MDSC in T cell homeostasis and maternal-fetal tolerance during pregnancy.

Transient hypogammaglobinemia of infancy (THI) is a group of disorders in early childhood characterized by decreased levels of immunoglobulins, specifically IgG (30). While THI is self-limited with IgG levels returning to normal by age 2–6 years of age, it places infants at higher risk for infections, e.g., pneumonia, digestive tract infections, or meningitis. While the underlying mechanism is still unclear, data have demonstrated a potential role of increased regulatory T cells (Tregs) in this patient population (30). Because MDSC induce expansion and activation of Tregs, a recent study examined their role in THI (30). In this small, retrospective study, 16 patients with THI were found to have increased G-MDSC compared to healthy controls. Additionally, there was a positive correlation between G-MDSC and the number of Tregs in children with THI (30). It is therefore possible that an aberrant persistence of MDSC after birth may contribute to the expansion of Tregs and mediate THI in infants, making MDSC a potential therapeutic target that could help children with THI mount more robust antibody responses when encountering infections.

## Oncology

### B-cell acute lymphoblastic leukemia

In agreement with previously published research in adults, the data from pediatric oncologic studies suggest that MDSC-mediated immunosuppression is associated with worse outcomes in children with cancer. For example, B-cell Acute Lymphoblastic Leukemia (B-ALL) is the most common cancer in children in the United States (31). While underlying genetic mutations are a major driver in the development of B-ALL, a dysregulated immune response also contributes by allowing for cancer cell evasion and proliferation (15). MDSC are an important component of this dysregulated immune response that leads to B-ALL in children. In 2016, Liu et al. demonstrated the expansion of G-MDSC in 43 pediatric patients with B-ALL (15). Peripheral blood mononuclear cells (PMBC) and bone marrow samples were collected from patients with B-ALL before and after 29 days of induction therapy (35 children achieved remission, 8 did not). Compared with 21 age-matched controls, G-MDSC were significantly increased in all patients prior to treatment in both the bone marrow and the peripheral blood. In the 35 children that achieved remission, G-MDSC decreased to numbers comparable to healthy controls. However, in the 8 subjects that did not achieve remission, G-MDSC numbers remained high post-induction. The study also found a positive correlation between the percentage of G-MDSC and minimal residual disease (MRD), a valuable prognostic factor representing the presence of post-therapeutic leukemia cells that increase the risk of relapse. The rise of G-MDSC during B-ALL treatment was associated with increased risk of MRD and indicated a negative prognostication (15). Decreased G-MDSC ex vivo led to enhanced proliferation of both CD4 + and CD8+ T cells and MDSC isolated from patient blood who achieved remission were not able to suppress T cells. Investigation into the mechanism of G-MDSC induced T cell suppression demonstrated increased expression of the STAT3 signaling pathway and ROS in patients with B-ALL compared to healthy controls, supporting this pathway as a key suppressive mechanism. Additionally, G-MDSC from B-ALL patients significantly impaired NK cell function, further supporting the immunosuppressive effects of G-MDSC and implicating these cells as a potential therapeutic target in children with B-ALL (15).

Beyond suppression of T and NK cells, emerging data also supports that MDSC induce expansion of immunosuppressive Tregs in children with B-ALL (32). In 31 children newly diagnosed B-ALL patients, compared to 27 age- and sex-matched healthy controls, results showed a significant expansion of G-MDSC and Tregs with a concurrent reduction in CD4+ T cells (32). MDSC and Treg numbers directly correlated with the levels of peripheral and bone marrow blasts present following induction. Moreover, complete remission was associated with a reduced percentage of G-MDSC and Tregs from pre-induction baselines that approached levels seen in the healthy children. These findings further implicate MDSCs in immune suppression during B-ALL progression and suggest that increased MDSCs and Tregs may serve as independent predictors of disease severity and could represent a targeted therapy approach as adjuvant treatment in chemotherapy regimens (32).

### Neuroblastoma

Among childhood solid tumors, MDSC have been best studied in pediatric neuroblastoma, a tumor of the sympathetic nervous system and one of the most common solid tumor malignancies in children (33). Chimeric antigen receptor (CAR) T cell therapy is a rapidly evolving therapeutic option for B-cell malignancies that are refractory to conventional therapy. However, efficacy in refractory solid tumors, including neuroblastoma, is limited. Tumino et al. investigated the presence of MDSC as a potential impediment to the success of CAR-T cell therapy in refractory neuroblastoma (34). Children with relapsed neuroblastoma malignancy after CAR-T cell therapy have increased levels of G-MDSC in peripheral blood (34). *in vitro* studies indicate that G-MDSC inhibit the anti-tumor cytotoxicity of the CAR T-cells therapy, and CAR T-cells conditioned with G-MDSC show downregulation of genes involved in cell activation, transduction, inflammation, and cytokine production (34). Similar to chemotherapy regimens for adult solid tumors, these data suggest that targeting MDSC may be a beneficial adjuvant therapy to CAR T-cells for children with high-risk, refractory neuroblastoma.

### Infection

Infection triggers a pro-inflammatory response to eliminate the pathogen while simultaneously activating a counterregulatory anti-inflammatory response to mitigate damage from unnecessary inflammation. MDSC expansion occurs as part of this compensatory anti-inflammatory response. However, some pathogens take advantage of this mechanism to suppress immune responses to promote their survival and replication (8). An example of this pathophysiology is in patients with chronic lung inflammation due to cystic fibrosis (CF). Patients with CF are often colonized with bacterial pathogens and are at higher risk for invasive infection, particularly those caused by *Pseudomonas aeruginosa (*35). Children with CF and chronic pseudomonal infections have significantly increased percentages of MDSC, specifically G-MDSC, in peripheral blood compared to age-matched healthy children without underlying lung disease. Furthermore, in children with CF, an increased percentage of G-MDSC was associated with worse obstructive pulmonary function (35). *in vitro* studies demonstrate that *Pseudomonas* bacteria induce MDSC differentiation via the flagellin protein and that MDSC isolated from CF patients with chronic *Pseudomonas* infection potently suppress healthy T cells in co-culture (35, 36). Targeting MDSC may allow for improved host responses to help children with CF more effectively clear these infections.

Increased MDSC are also observed in children with other types of lung infections. For example, children with recurrent infections have increased numbers of M-MDSC in peripheral blood correlating with a decreased frequency of CD8+ T cells (18). In a co-culture experiment using healthy T cells, MDSC from children with recurrent respiratory tract infections demonstrated higher expression of Arg-1 and were more suppressive of T cell proliferation and function than MDSC from children without recurrent infections (37). Similar findings occurred in children with acute SARS-CoV-2 infection. In a study of 80 children, those hospitalized with SARS-CoV-2 had increased MDSC, particularly G-MDSC, compared to healthy children (38). Additionally, the increased percentage of MDSC in hospitalized children with COVID-19 was associated with an increased need for respiratory support and a longer length of stay (38). MDSC are also implicated in the pathophysiology of tuberculosis (TB) (39). TB remains a global healthcare crisis and children infected with human immunodeficiency virus (HIV) are at high risk of developing TB after exposure (40). Evidence suggests that MDSC frequency in peripheral blood is significantly elevated in children exposed to TB, regardless of HIV infection status, compared to those without TB. Additionally, MDSC frequencies, particularly M-MDSC, were higher in children with active TB infection compared to children with household exposure to TB that did not develop active infection (40).

A growing body of evidence suggests that a subset of children with septic shock can develop an exaggerated anti-inflammatory response that is associated with an increased risk of nosocomial infection and mortality (41, 42). MDSC may be important contributors to immune impairment in these children given their potent suppression of T cells. A prospective, observational study demonstrated an increased percentage of MDSC, particularly the G-MDSC subset, in children with septic shock compared to age-matched, healthy controls (43). The frequency of MDSC peaked in the first 48 h of admission and ROC curve analysis determined that a cut-off point of 25% MDSC of peripheral blood mononuclear cells was predictive of prolonged organ dysfunction in septic children (43). Multisystem inflammatory syndrome in children (MIS-C) is characterized by a hyperinflammatory syndrome that can occur 2–6 weeks following SARS-CoV-2 infection and, similar to septic shock, is due to a dysregulated immune response (44). Children with MIS-C admitted to the ICU have a higher percentage of MDSC and significantly decreased numbers of CD4 + and CD8+ T cells compared to those admitted to the ward. Additionally, the expansion of MDSC persists for several weeks in the children who were critically ill due to MIS-C (44). Although MDSC help temper the inflammatory response and mitigate damage to healthy tissue, their persistence may contribute to delayed pathogen clearance and continued dysregulated immune responses in septic shock and MIS-C. Future studies investigating when MDSC are harmful during disease processes is necessary to determine the potential timing of therapeutic interventions targeting this cell population and designing clinical trials.

### Atopic diseases

Atopy is characterized by skewing of T cell subsets to favor the predominance of T helper 2 (Th2) cells and includes diseases such as asthma and allergic rhinitis. Studies investigating the role of MDSC in the pathophysiology of asthma demonstrate that children with asthma have increased MDSC compared to healthy controls (45, 46). One study compared children during an asthma exacerbation to a healthy control group, a group of children with pneumonia and a group of children whose asthma had been alleviated with budesonide, an inhaled steroid therapy (46). The asthma exacerbation group was found to have elevated levels of MDSC that were associated with increased levels of IL-10, an anti-inflammatory cytokine. Prior studies indicate that IL-10 inhibits the onset of asthma through decreased Th2 cytokine production (47). In contrast, a different study demonstrated that increased MDSC in children with asthma compared to those with pneumonia or healthy controls was associated with increased Th17 cells, a proinflammatory T cell subset that contributes to airway inflammation (45). It is unclear if MDSC contribute to asthma exacerbations or if their expansion can help temper airway inflammation and shorten exacerbation periods.

Like asthma, allergic rhinitis is another common pediatric condition where T cell responses are implicated in the underlying pathophysiology, specifically an exaggerated Th2 response. In one study, sixty children with allergic rhinitis were compared to fifty healthy controls (48). The percentage of MDSC, particularly M-MDSC, in peripheral blood was significantly elevated in the allergic rhinitis group compared to the healthy controls and correlated with increased expression of CD28, a driver of allergen-specific Th2 responses. Although, collectively, these studies demonstrate an association between MDSC and asthma or allergic rhinitis, it is not clear if MDSC are harmful in these circumstances. More research is needed to completely understand the complex role of MDSC in pediatric autoimmune diseases.

### Pulmonary hypertension

Pulmonary hypertension (PH) is characterized by worsening pulmonary vascular resistance that leads to right-sided heart failure and is associated with high morbidity and mortality (49). Patients with PH demonstrate changes in the thickness of the walls of the pulmonary arteries and perivascular accumulation of cells from both the innate and adaptive immune system, including dysregulate T cell subsets (50). A study in 2012 quantified peripheral MDSC in 26 children with pulmonary hypertension compared to 10 healthy controls. Patients with PH had a higher frequency of circulating MDSC compared to the healthy controls, and higher numbers of MDSC correlated with increasing mean pulmonary artery pressure (51). This study also demonstrated an increase in Arg-1, an important mechanism of MDSC-mediated T cell suppression (51).

### Type 1 diabetes

DM1 is defined by T cell destruction of pancreatic, insulin-producing beta cells (52).

Studies have also demonstrated expansion of MDSC in type 1 diabetes mellitus (DM1) (52, 53). Studies suggest that MDSC may have a beneficial role in T cell suppression in DM1, slowing the beta cell destruction and delaying the onset of DM1. Initial murine models demonstrated an expansion of MDSC in the DM1 phenotypic mouse in peripheral blood but a decrease in MDSC in islet cells (52). This group demonstrated that MDSC suppressed T cells and decreased the risk of DM1 in mice. In human studies, M-MDSC are expanded in the peripheral blood of patients with DM1 when compared to healthy controls. A functional suppression assay demonstrated that MDSC from subjects with DM1 suppressed T cells in a contact-dependent manner (52). Intriguingly, the suppressive T cell activity observed in DM1 patients was less suppressive than *in vitro* generated MDSC (52), suggesting that endogenous MDSC suppressive function may be improved by cytokine stimulation to impede development and progression of DM1.

### Vaccine efficacy

Vaccinations have made a tremendous impact on public health and are estimated to prevent at least 146 million deaths in children less than 5 years of age since their development (54). T cells play an important role in vaccine efficacy and impaired T cell responses can result in failed immunity (55). MDSC-mediated T cell suppression is hypothesized to contribute to impaired vaccine responses. In a murine model of the *Mycobacterium bovis* Bacillus Calmette-Guerin (BCG) vaccine, MDSC were recruited to local tissues at the site of vaccination and dampened T cell response via increased production of Arg-1, facilitating pathogen persistence (56). Cancer targeted vaccines are an important adjuvant therapy in chemotherapy regimens, and studies in adults with cancer indicate that inhibition of MDSC augment the efficacy of cancer vaccines and improve outcomes (57). In a South African study of 91 healthy infants, increased MDSC was not associated with impaired responses to routine vaccines, including Hepatitis B, *Haemophilus influenzae* B, and Diphtheria, Tetanus, acellular Pertussis (58). However, there may be specific patient populations, such as children with human immunodeficiency virus (HIV) and increased MDSC, where MDSC inhibition may augment vaccine strategies.

## Discussion

Compared to the extensive characterization of MDSC in adult populations, our understanding of these cells in children remains very limited. This review summarizes the small but growing body of evidence suggesting that MDSC play an important role in a wide variety of pathologies in pediatric patients. In adults, MDSC are well-characterized as critical mediators of immune suppression, promoting tumor growth and inhibiting T-cell responses. Studies have extensively detailed the signaling pathways, phenotypic markers, and mechanisms of immune modulation by MDSC in adults. Conversely, research on pediatric MDSC is significantly limited with a greater focus on identifying the presence of MDSC in the neonatal period and during acute infection in children. Critical gaps remain in understanding the molecular and functional distinctions of MDSC across the pediatric age spectrum, particularly how MDSC change as a child's immune system matures and adapts.

MDSC exhibit a dichotomy in function, often straddling protective and pathological roles. In neonates, MDSC help establish immune tolerance and support the development of the gut microbiome, which are processes essential for avoiding harmful inflammation fostering immune system development. However, their persistence or overactivation can contribute to severe infections causing neonatal sepsis, an important cause of morbidity and mortality in these fragile patients. Similarly, in pediatric autoimmune diseases: their capacity to suppress pro-inflammatory responses may alleviate hyperinflammation and tissue damage, but this same suppressive function can create an immunosuppressive environment for tumors to exploit. In pediatric cancers such as B-cell acute lymphoblastic leukemia (B-ALL) and neuroblastoma, tumor cells utilize MDSC to evade immune surveillance, promoting tumor progression and resistance to therapies. Understanding this duality is crucial for developing strategies to modulate MDSC activity appropriately in pediatric settings, aiming to harness their protective roles while mitigating their pathological contributions.

The therapeutic potential of targeting MDSC in children is both promising and challenging. On one hand, inhibiting MDSC could improve T cell and NK cell responses to allow the host to better target cancerous tissues, enhancing outcomes in immunotherapy and chemotherapy. Conversely, augmenting MDSC function might benefit patients with autoimmune diseases or severe inflammatory conditions, such as graft vs. host disease. Pediatric patients also present unique challenges, such as developmental variations in the prevalence and function of different immune cell populations. These differences complicate the translation of adult-derived therapies into pediatric care. Future studies are needed to confirm the mechanisms used by MDSC to induce immune suppression in children and better understand circumstances in which MDSC are harmful or helpful to determine which patients would potentially benefit from therapies targeting MDSC.

### Future directions

• Function of MDSC in immune homeostasis and development: Infants and young children experience rapid growth and development in their early years, including evolution of their immune system. Understanding the function of MDSC in immune homeostasis along the continuum of development is a much-needed area of research. Published studies demonstrate that MDSC are necessary for neonates as they adjust to life ex utero, but we do not fully understand at what age the persistence of higher frequencies of MDSC becomes pathologic.

• Identification of unique cellular surface markers: Additionally, MDSC do not have unique cell surface markers and confirming their ability to suppress T cells remains a part of their definition. Isolating MDSC for co-culture experiments is particularly challenging in pediatric studies given the limits on blood volume that can be safely collected from young children. The identification of markers unique to MDSC would significantly improve investigators’ ability to further characterize these cells in children using smaller blood samples.

• Mechanisms of MDSC-mediated suppression in children. Mechanisms of MDSC-mediated suppression are well characterized in adults with various malignant tumors but very little is known about mechanisms employed by MDSC in children with different pathologies. Although Arg-1, PD-L1 and ROS are key pathways employed by MDSC in adults, MDSC in children may suppress T cells *via* other pathways.

• Translating from bench to bedside: The dichotomous nature of MDSC means they can have beneficial or harmful effects for different pathologies, and at different times points during disease. MDSC activation and suppression may also vary by compartment, e.g., peripheral blood vs. lung tissue. Understanding when MDSC need to be targeted in specific patient populations and choosing the appropriate immunotherapies, e.g., PD-L1 inhibitors, are necessary to provide more precise and personalized medicine to children.
